# Beyond scalar metrics: functional data analysis of postprandial continuous glucose monitoring in the AEGIS study

**DOI:** 10.1186/s12874-025-02748-2

**Published:** 2026-01-24

**Authors:** Marcos Matabuena, Joseph Sartini, Francisco Gude

**Affiliations:** 1 Biostatistics Dept., Harvard University, 677 Huntington Ave, Boston, MA 02115 USA; 2 Present Address: Mohamed bin Zayed University of Artificial Intelligence, Abu Dhabi, UAE; 3 Biostatistics Dept., Johns Hopkins University, 615 N Wolfe St, Baltimore, MD 21205 USA; 4 Dept. of Medicine, Universidad de Santiago de Compostela, Praza do Obradoiro, Santiago de Compostela, 15705 Spain; 5 Research Methods Group (RESMET), Health Research Institute of Santiago de Compostela, Santiago de Compostela, Spain

**Keywords:** Continuous glucose monitoring, Postprandial glucose, Functional data analysis, Glucose metabolism, Hierarchical modeling

## Abstract

**Purpose:**

Postprandial glucose, collected through continuous glucose monitoring (CGM), has established clinical relevance in assessing metabolic capacity and informing diet prescriptions. However, most studies of postprandial glucose summarize these data into scalar values, such as 2-hour area under the curve (AUC) or 2-hour peak glucose. We propose analyzing the full CGM time-series trajectories to provide more detailed insights. Given the smooth dynamics of glucose metabolism, the resulting data are inherently functional, with hierarchical structure when there are multiple time series per participant.

**Methods:**

We consider multilevel functional data analysis (FDA) techniques to analyze postprandial CGM trajectories, applying these methods to data from participants without diabetes in the AEGIS study. The AEGIS study collected meal timing and nutrient composition during periods the participants wore CGM devices. We illustrate the utility of FDA methods to characterize postprandial CGM variability and to explore the associations between dietary/patient characteristics and CGM over the postprandial period. We introduce an extension of the R-squared ($R^2$) metric to hierarchical functional models to quantify variability explained in this context.

**Results:**

The FDA models indicate that, for many nutrients, the effect of dietary composition varies throughout the 6-hour post-prandial temporal window. For example, fiber blunts the postprandial glucose response 90 minutes after the meal, while fats reduce the response during the first 50 minutes. In addition, metabolic responses to dietary intake differ between normoglycemic and prediabetic individuals as expected.

**Conclusion:**

Analyzing postprandial glucose responses with functional methods yields temporal insights that traditional scalar approaches cannot capture. Stratifying the analysis by glycemic status (normoglycemic vs. prediabetes) also provides novel findings.

## Background

While there is substantial literature regarding the impact of meal composition on scalar summaries of the postprandial glucose response (PPGR), few studies have examined how the continuous glucose time series during the postprandial window is impacted by diet [1–4]. The Estrada Glycation and Inflammation Study (AEGIS) provides an opportunity to examine this relationship in a free-living setting through the concurrent collection of meal timing and continuous glucose monitoring (CGM) data. Recent advances in wearable technology and smartphones have revolutionized the collection of physiological time series data in large samples [5–7], and continuous glucose monitoring is no exception [8–15]. CGM captures quasi real-time glycemic response data helpful for diabetes management, screening, and general assessment of glucose metabolism [12, 16, 17]. A limited number of studies have attempted to holistically characterize time-dependent metabolic response patterns in postprandial CGM time series, most focusing on specific features such as peaks [18]. Many prior nutritional studies focus primarily on metabolites [19], collect serum blood glucose at fixed time points after meals [20], or summarize CGM data into simple scalar summaries such as 2-hour AUC, 2-hour peak glucose, or 2-hour mean glucose [21–23]. These scalar summaries of CGM responses have been fundamental to understanding metabolic control. Recent work by [24] emphasizes the essential CGM metrics required for effective diabetes management, particularly in evaluating the glycemic response to different meals and over time periods. Analysis of the entire postprandial glucose response trajectories ensures that no clinical information is lost during aggregation.

CGM devices facilitate characterizing how postprandial glucose trajectories vary by diet and individual. Care must be taken to appropriately model the corresponding glucose data. Differential equation (DE) frameworks based on physiological principles have been proposed to model these data, but these systems often require observing the concentrations of related hormones glucagon and insulin [25–29]. Alternatively, one can leverage the rich literature of DE-based minimal models governing just glucose dynamics [30–32]. Although physiologically well–motivated, these models can be computationally demanding and often provide no way to estimate time–dependent associations between predictors and the glucose response. These problems are compounded when the data have a hierarchical structure (e.g., multiple postprandial glucose trajectories per individual).

Analyzing postprandial glucose over time using CGM is an accurate and comprehensive way to assess metabolic capacity for metabolically-healthy individuals. Several machine-learning studies have recently addressed this topic [33–35]. However, these studies focus mainly on combining scalar CGM summaries and non–CGM biomarkers.

**Fig. 1:**
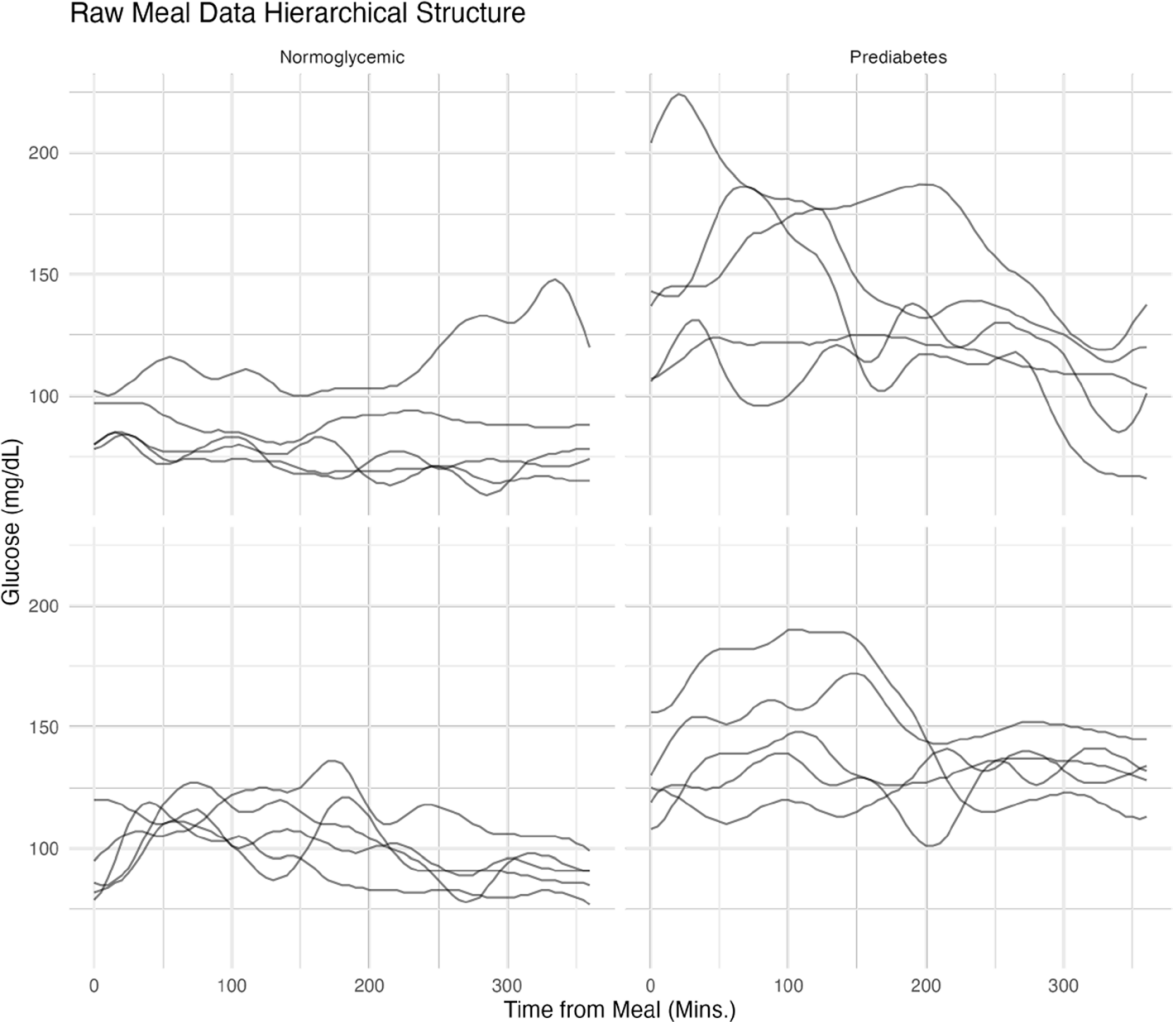
Postprandial CGM data over several days in four individuals. Each panel corresponds to one participant. Those in the first column are normoglycemic, and those in the second column have prediabetes. Glycemic status was established according to the American Diabetes Association (ADA) criteria based on glycated hemoglobin (HbA1c) and fasting plasma glucose (FPG) values

Functional Data Analysis (FDA) [36] is a branch of statistics that provides methods for analyzing mathematical functions instead of, or together with, scalar values. Therefore, FDA is a natural analytical framework for time-dependent postprandial glucose trajectories produced by CGM devices. FDA techniques can mitigate the analytical limitations that arise when CGM data are reduced to a small set of discrete summaries and can improve our understanding of postprandial glycemic responses through models that preserve the continuous–time nature of the input data [37–39]. To gain a deeper understanding of the need to use FDA methods, Fig. 1 illustrates glucose response trajectories after meals for four individuals, two normoglycemic and two with prediabetes, under free-living conditions over six days. This Figure highlights the substantial inter-individual and inter-day variability observed over the 6-hour post-meal period. Our goal is to partition this variability into diet-related effects and residual (unexplained) variation. The two pre–diabetic participants show greater day-to-day fluctuations, whereas the four normoglycemic individuals display more consistent patterns. FDA has already emerged as a promising approach for a variety of CGM-data modeling tasks, from analyzing glucose distributions to characterizing nocturnal glycemic patterns [11, 12, 15, 40–43]. However, comprehensive examples detailing the application of multilevel functional modeling to postprandial CGM data are limited, despite the natural applicability of FDA methods in this context

This article provides practitioners with a step–by-step guide for analyzing postprandial CGM data, with the goal of fostering the broader adoption of these statistical techniques in similar datasets and scientific problems. We demonstrate how FDA methods can be applied directly, using existing software, to efficiently estimate variability modes in glucose trajectories and to quantify associations between participant characteristics, meal composition, and glycemic response trajectories.

We proceed by providing details of the AEGIS study, explaining the FDA methodologies, and finally demonstrating the results of applying these methods to hierarchically structured postprandial CGM data from AEGIS. We focus first on descriptive analyzes, examining the modes of variability in postprandial CGM responses using Multilevel Functional Principal component analyzes (MFPCA). Then, we investigate statistical associations between covariates, including participant characteristics and meal composition, and postprandial CGM response curves using Function-on-Scalar Regression (FoSR). For both MFPCA and FoSR, it is of interest to understand how much variability the model captures. For MFPCA, explaining a low amount of variability is a good diagnostic indicator that the model structure or truncation is potentially ill-suited to the data. In FoSR, quantifying the variability explained by the selected predictors is essential both for model selection and for interpreting inferences in a broader context. Since this is still an open problem in FDA, we conclude by proposing an extension of marginal and conditional $R^{2}$ to hierarchical functional models.

**Fig. 2:**
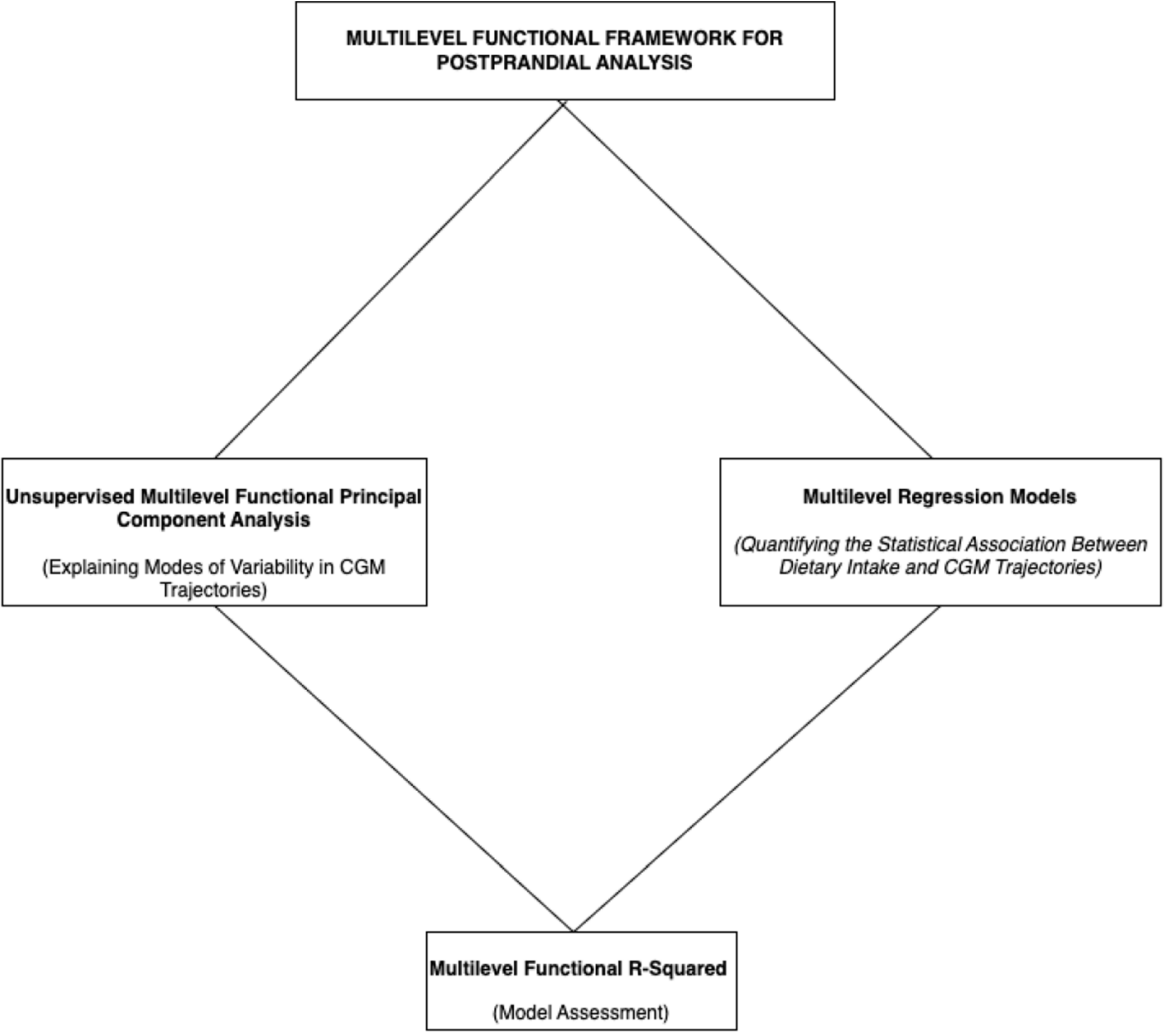
Pipeline steps for a multilevel postprandial framework

Figure 2 summarizes the core steps of the pipeline for analyzing postprandial glucose data using FDA.

## Data: the a Estrada Glycation and inflammation study (AEGIS)

### Objective and design

The A Estrada Glycation and Inflammation Study (AEGIS; trial registration NCT01796184 [registration date: 2013-02-14]) was a ten-year longitudinal study investigating changes in blood glucose and their associations with inflammation and obesity [44]. The study examined the relationships between these factors and the potential development of comorbidities such as diabetes mellitus. AEGIS involved a stratified random sample of individuals 18 years of age and older drawn from the Spanish National Health System Registry. The trial also collected CGM for a subsample of the study population, providing detailed glucose profiles at various time points over a 5-year period.

At the beginning of the study, a random sample from the general population of 1,516 individuals underwent extensive medical examinations to construct detailed clinical profiles. Diaries, laboratory biomarkers, and questionnaires were used to assess metabolic capacity, mental well-being, and lifestyles. Table 1 summarizes all relevant scalar predictors used in our analyzes.

**Table 1:** Description of the variables used collected by AEGIS. Summaries of continuous variables include Mean (Standard Deviation), then Median [Min., Max.]

Variable	Distribution summaries	
	Normoglycemic ($N=319$)	Prediabetes ($N=58$)
Individual Level^a^		
Age (yrs)	44.6 (13.7) 44.0 [18.0, 81.0]	58.7 (12.0) 61.0 [23.0, 84.0]
Weight (kg)	73.7 (14.3) 72.5 [41.0, 130]	83.0 (19.6) 79.2 [49.0, 145]
Gender	Male: 121 (37.9%) Female: 198 (62.1%)	Male: 18 (31.0%) Female: 40 (69.0%)
HbA1c (%)	5.25 (0.25) 5.30 [3.10, 5.60]	5.86 (0.20) 5.80 [5.70, 6.40]
Meal Level^b^		
Carbohydrates (g)	59.9 (40.5) 52.3 [0, 513]	53.7 (37.5) 45.7 [0, 226]
fats (g)	30.1 (23.8) 25.4 [0, 237]	25.7 (22.3) 21.8 [0, 169]
Proteins (g)	27.5 (17.9) 24.2 [0, 200]	25.9 (17.0) 23.1 [0.4, 105]
Fiber (g)	8.8 (6.7) 7.2 [0, 89.3]	9.1 (7.0) 8.1 [0, 63.1]
Initial CGM Glucose (mg/dL)	103 (15.3) 101 [52, 237]	110 (19.4) 107 [56, 196]

^a^All individual-level covariates collected at screening

^b^All dietary information assessed through self-report and dietitian reconstruction

### CGM and nutrition protocols

CGM data were collected for a subset of 581 participants in a two-sample design, including 516 individuals without diabetes. The remaining 65 individuals were identified as having previously undiagnosed diabetes mellitus and were subsequently excluded. Of the remaining participants, 377 recorded at least one meal that they labeled as “dinner”. Our analyzes focus on this subset and meals denoted as “dinner” for the sake of consistency. For each such meal, we extracted the 6 hours of CGM data after the reported meal time to construct postprandial glucose trajectories.

The participants were fitted with Enlite™ sensors and iPro™ CGM devices, offering blinded interstitial glucose measurements every 5 minutes for up to seven days. On the seventh day, the sensor was removed, and data were downloaded for analysis. Specifics on CGM placement and calibration can be found in [44].

Throughout the 7-day CGM monitoring period, participants were instructed to maintain their usual routines while recording every eating occasion in a food diary spanning the same midnight-to-midnight window as the sensor. For each meal or snack, they noted the clock time, portion size, preparation method (e.g. grilled, fried, baked), ingredients, and any sauces or condiments. At the end of the week, a registered dietitian reviewed each diary face-to-face with the participant to clarify ambiguities, fill in omissions and, when necessary, quantify portions with a validated photographic atlas of household measures. Only meals that could be fully and reliably decomposed were retained. The final journals were coded in Dietowin® 8.0 (Biologica–Tecnologica Médica, Barcelona, Spain), producing daily totals for energy (kcal); macronutrients (carbohydrate, sugar, fiber, protein, total fat and its saturated, monounsaturated, and polyunsaturated fractions); cholesterol; and minerals Ca, Fe, Mg, P, K and Na.

The CGM data were processed conservatively to ensure data quality. The entire first 24 h of wear—during which the sensor accuracy was markedly lower (MARD $\approx$ 12%)—was discarded. Any subsequent 24-hour segment with more than two consecutive hours of signal loss was excluded, thus removing compression artifacts and extended drop-outs. Each remaining day had to contain at least three capillary finger-stick values for calibration; days that did not meet this requirement were removed without numerical imputation. These rules, together with daily calibrations, limited inter-day drift.

The analyses in the present paper focus on evening meals (dinners). Under free-living conditions, dinner is the meal least affected by daytime activity and environmental variability, providing a clearer metabolic signal and greater comparability between individuals. Although the modeling framework can be applied to any meal of the day, our study design and data-quality criteria make dinner the most reliable context for evaluating postprandial glucose dynamics.

In our analysis, we focused on a 6-hour period after dinner. Although a 6-hour post-prandial window is not standard practice, our functional modeling framework captures glucose responses over the entire period. This extended window naturally encompasses the conventional 2- and 3-hour intervals, while also capturing longer-term metabolic responses and their statistical associations.

## Methods

### Notation

The primary outcomes of interest are the hierarchical postprandial CGM curves. We assume that these curves are noisy, discrete observations of some underlying function $Y_{ij}(t)$. We consider the index $i \in \{1,\ldots , n\}$ to be the participant - *n* subjects in total. The next index $j \in \{1,\ldots , J_i\}$ indicates the day of observation, where the number of days recorded is $J_i$ for participant *i*. Although standard FDA methods do not require that each participant have the same number of recorded meals – for example the AEGIS data set contains heterogeneous counts $J_i$ – we assume a common value $J_i = J$ between all individuals for the mathematical explanation of this section. Finally, we consider the domain $t \in [0,360]$, which represents the time in minutes since the reported mealtime. In practice, each function is observed only at a finite set of time points, $t \in T_m = \{t_{0}=0,\ldots ,t_{m}=360\}$.

### Multilevel functional principal components analysis

Consider the definition of the MFPCA model in Eq. 1, as initially proposed in [45]. For each $t\in [0,360]$, $\mu (t)$ is the global mean, $\mu (t)+\nu _{j}(t)$ is the mean response for meal/day *j*, $U_{i}(t)$ is the subject-specific deviation from the meal-specific mean function, and $W_{ij}(t)$ is the subject- and meal-specific deviation from the subject-specific mean. Here, $\mu (t)$ and $\nu _{j}(t)$ are assumed to be fixed functions. Although we do not expect there to be any day-specific mean shift $\nu _j(t)$, as there is no consistent pattern in meals taken by day, we include this term as standard. We assume that $U_i(t)$ and $W_{ij}(t)$, on the other hand, are random functions with expectation $\mathbb {E}[U_i(t)] = \mathbb {E}[W_{ij}(t)] = 0$.

1 $$\begin{aligned}Y_{ij}(t)&= \mu (t)+\nu _{j}(t)+ U_{i}(t)+W_{ij}(t)\\& \quad \forall i \in \{1,\ldots , n\}, j \in \{1,\ldots , J\}, t\in [0,360]. \end{aligned}$$

MFPCA proceeds by decomposing $U_i(t)$ and $W_{ij}(t)$ to characterize variability at two hierarchical levels: between and within-subjects. Doing so relies on the functional analog of principal component analysis (PCA). We decompose $U_i(t)$ and $W_{ij}(t)$ in the way exhibited by Eq. 2, where $\phi _{k}(t)$ for $k \le K$ and $\psi _{h}(t)$ for $h \le H$ are orthogonal eigenfunctions and $a_{ik}$/$b_{ijh}$ the associated scores or scalar weights.

2 $$\begin{aligned} U_i(t)= & \sum \limits _{k=1}^{K} a_{ik} \phi _{k}(t) \hspace{0.2cm} \forall i \in \{1,\ldots , n\}, \nonumber \\ W_{ij}(t)= & \sum \limits _{h = 1}^{H} b_{ijh} \psi _{h}(t) \hspace{0.2cm} \forall i \in \{1,\ldots , n\}, j \in \{1,\ldots , J\}. \end{aligned}$$

We assume that the scores are normally distributed with mean zero and variance equal to the eigenvalues associated with each eigenfunction. As in standard PCA, we order the eigenfunctions at each level so that they explain decreasing amounts of the observed variability at their respective levels. These eigenfunctions correspondingly indicate the modes of variability in the observed data at each level. Further, they provide a parsimonious method to represent the observed data: using the corresponding low-dimensional set of scores.

There is accessible software to estimate the components of the MFPCA model using replicates of data $Y_{ij}(t)$ that are discretely observed over an interval ($t \in T_m$). The refund package provides an efficient method to estimate MFPCA using the mfpca.face function [46]. This software uses bivariate penalized splines to produce estimates which are robust both to different numbers of observed meals per person and to sparsity in the domain along which the functions are observed.

In this study, we focus on describing the diverse modes of variability in the glucose trajectory in terms of eigenfunctions and eigenvalues, facilitating a comprehensive understanding of the data structure. We choose the number of eigenvalues *K*, *H* at both levels here using our extended $R^2$ measure of the explained variability. We estimated this value over a range of *K*/*H* values in Supplement 6, where we see a slight elbow in $K = H = 3$, the hyperparameter chosen for our analyzes.

### Predicting clinical outcomes using latent representations

After fitting the models discussed in Function-on-scalar regression section, we used the residuals to define latent clusters at the individual level. In the next subsection, we outline the method used to compute these residuals.

Consider an individual $i \in \{1,2\ldots ,\ n\}$, and the corresponding repeated observations $j\in \{1,\ldots ,J\}$. The functional residual for each individual and observation was defined according to Eq. 3, where $Y_{ij}(t)$ denotes the observed glucose levels at postprandial time $t\in [0,360]$, and $\widehat{Y}_{ij}(t)$ is the estimated glucose level using only the fixed effects in the multilevel regression model, as detailed in Eq. 4.

3 $$\begin{aligned} \widehat{\epsilon }_{ij}(t) = Y_{ij}(t) - \widehat{Y}_{ij}(t) \end{aligned}$$

4 $$\begin{aligned} \widehat{Y}_{ij}(t) = \sum \limits _{l=1}^L X_{ij,l}\widehat{\beta }_l(t) \end{aligned}$$

As $\widehat{\epsilon }_{ij}(t)$ are random functions in $L^2([0,360])$, the MFPCA methodology described in Multilevel functional principal components analysis section is applicable to these residuals. This facilitates both the characterization of functional residuals and the summarization of them into vectors of eigenfunction scores that could be used in the prediction of clinical outcomes. We modeled the residuals $\widehat{\epsilon }_{ij}(t)$ using an Eq. 1–style formulation (Eq. 5).

Applying the same eigenfunction decomposition described in the MFPCA section to person-level functions $U_i(t)$, we obtain score vectors $\widehat{a}_i \in \mathbb {R}^K$. These scores succinctly summarize the information for each individual, with the associated eigenfunctions indicating the main types of variation present in the residual trajectories (e.g., stable, positive, or negative deviations). Given a scalar outcome $Z_i \in \mathbb {R}$ of interest (e.g., the HOMA-IR surrogate marker of insulin resistance), we regress this outcome on residual coordinates and additional individual-level characteristics using a fixed-effects model. Denoting the other covariates for the individual *i* by $X_i \in \mathbb {R}^p$, with components $X_{ik}$, we consider the general regression model in Eq. 6, where $\gamma _j$ and $\beta _k$ are regression coefficients and $\epsilon _i$ is an error term,

5 $$\begin{aligned}\widehat{\epsilon }_{ij}(t)&= \mu (t) + \nu _{j}(t) + U_{i}(t) + W_{ij}(t)\\& \quad \forall i \in \{1,\ldots , n\}, j \in \{1,\ldots , J\} . \end{aligned}$$

6 $$\begin{aligned} Z_i = \sum \limits _{j=1}^{m} \gamma _j \widehat{a}_{ij} + \sum \limits _{k=1}^{p} \beta _k X_{ik} + \epsilon _i \end{aligned}$$

### Function-on-scalar regression

FDA also includes Function-on-Scalar Regression (FoSR), a method for ascertaining associations between scalar predictors and a functional response. In addition to postprandial glucose $Y_{ij}(t)$, AEGIS collected covariates such as demographic data, HbA1c, and meal-level dietary information. We denote these scalar covariate values using $X_{ijl}$ where the index $l\in \{1,2,\ldots ,L\}$ refers to a particular covariate. Although this notation indicates that the covariates vary by both person and meal, it is important to note that person-specific features remain fixed over meals.

FoSR presupposes that the outcome function $Y_{ij}(t)$ can be written as the linear combination of the scalar covariates and coefficient functions $\beta _l(t)$. These $\beta _l(t)$ provide an estimate of the association between each predictor and $Y_{ij}(t)$ at time $t\in [0,360]$. One can view this as an extension of the basic linear model, where the scalar response and the coefficients are generalized to functions. Just as in a traditional linear model, we can extend FoSR to add functional random effects such that each participant has their own random deviation function. The form of the entire FoSR model, with subject-specific random-effects, can be found in Eq. 7.

7 $$\begin{aligned} Y_{ij}(t)= \sum \limits _{l=1}^L X_{ijl}\beta _l(t) + \alpha _{i}(t) + \epsilon _{ij}(t). \end{aligned}$$

Within this model, $\alpha _i(t)$ is a random functional effect corresponding to subject *i* at time *t*. This function plays a similar role to the person-specific deviation from MFPCA, denoted as $U_i(t)$. The function $\epsilon _{ij}(t)$ captures the residual variation that is unexplained by fixed or random effects. We assume that the $\alpha _i(\cdot )$ and $\epsilon _{ij}(\cdot )$ processes are zero mean and that $\epsilon _{ij}(\cdot )$ is uncorrelated with all $\alpha _i(\cdot )$.

Multilevel functional models such as the one in Eq. 7 can be fit efficiently using the R packages refund and fastFMM. The fosr() family of functions of the former package is best for moderately-sized data. These functions use flexible penalized-spline-based modeling to estimate both coefficients $\beta _l(t)$ and random effects $\alpha _i(t)$ using reduced rank regression [46]. This type of adaptive procedure is excellent at recovering the $\alpha _i(t)$, but can be computationally costly. However, if the sample size are large, the fui() function from fastFMM is better suited, as it leverages point-wise models to estimate the coefficients $\beta _l(t)$ [47]. More recently, scalable methods have been developed to estimate both coefficient functions $\beta _l(t)$ and random effect functions $\alpha _i(t)$ [48]. In our empirical analysis, we focus on the fui() methodology to reduce computational cost.

### $R^2$ for multilevel functional models

We extend the traditional notion of $R^{2}$ to multilevel functional models. This extension has two main objectives: (i) to assess how accurately the functional models can recreate the response over time, and (ii) to guide model selection so that the final model retains sufficient information for the analytical application.

$R^2$ is classical metric in statistical literature used to quantify the variance explained in a response variable by a set of corresponding predictors. For functional data, which are distributed approximately Gaussian point-wise, it is straightforward to extend the traditional formulae for $R^2$ to the functional case by evaluating them repeatedly in the functional domain.

We derive point-wise and global metrics $R^{2}$ for both MFPCA and FoSR. Our extension of $R^2$ takes advantage of the fact that both FoSR and MFPCA estimate the functional responses $Y_{ij}(t)$ at points $t\in T_m$ in the functional domain. For each $t\in T_m$, $i \in \{1, \ldots , n\}$ and $j\in \{1, \ldots , J\}$, we denote $\widetilde{Y}_{ij}(t)$ and $Y_{ij}(t)$ as estimated and observed functional trajectories, respectively. At any given time point $t \in T_m$, the point-wise $\widetilde{R}^2(t)$ of the supervised models can be estimated using the standard univariate approach as detailed in Eq. 8. Note that this formula includes averaging on both individual *i* and meal *j*, forming an aggregate estimate of variance explained over the entire data set.

8 $$\begin{aligned} \widetilde{R}^2(t)= & 1 - \frac{\sum _{i=1}^{n}\sum _{j=1}^{J} \left( Y_{ij}(t) - \widetilde{Y}_{ij}(t)\right) ^2}{\sum _{i=1}^{n}\sum _{j=1}^{J} \left( Y_{ij}(t) - \overline{Y}(t)\right) ^2}, \nonumber \\ \text {where} \ \overline{Y}(t)= & \frac{1}{nJ}\sum \limits _{i=1}^{n}\sum \limits _{j=1}^{J}Y_{ij}(t). \end{aligned}$$

We can produce estimates $\widetilde{R}^2(t)$ of the variability explained by MFPCA when including just the components at the participant-level and the whole model. For the participant-level, we form the predicted trajectories $\widetilde{Y}_{ij}(t)$ using the MFPCA estimates at only the first level of the hierarchy. To be specific, we set $\widetilde{Y}_{ij}(t)=\widehat{\mu }(t)+\widehat{\nu }_j(t)+\sum _{k=1}^{K}\widehat{a}_{ik}\widehat{\phi }_k (t)$ for estimated eigenfunctions at the participant-level $\widehat{\phi }_k(t)$ and scores $\widehat{a}_{ik}$. For the whole model, we correspondingly form the predicted trajectories using all estimates: $\widetilde{Y}_{ij}(t)=\widehat{\mu }(t)+\widehat{\nu }_j(t)+\sum _{k=1}^{K}\widehat{a}_{ik}\widehat{\phi }_k(t) + \sum _{h = 1}^H \widehat{b}_{ijh}\widehat{\psi }_h(t)$. In both cases, we can then apply Eq. 8 to get estimate $\widetilde{R}^2(t)$ of the point-wise $R^2$.

For FoSR models, we extend the marginal and conditional notions of $R^2$ introduced by [49]. This extension follows directly from the definition of these quantities in the original context of linear mixed effects models. The marginal $\tilde{R}^2(t)$ estimates the variability explained by fixed factors only, the $\sum _{l = 1}^L X_{ij,l} \beta _l(t)$ component of Eq. 7. We can leverage Eq. 8, setting $\widetilde{Y}_{ij}(t)$ equal to this quantity, to derive the marginal point-wise $\widetilde{R}^2(t)$. The conditional estimate will incorporate both fixed and random factors: covariate effects and person-specific deviation $\sum _{l = 1}^L X_{ij,l} \beta _l(t) + \alpha _i(t)$ from Eq. 7. The same procedure can be used to derive the conditional point-wise $\widetilde{R}^2(t)$. The respective fitted values used to estimate $R^2$ in each case are detailed in Eq. 9, using the covariates introduced for Eq. 7.

9 $$\begin{aligned} \widetilde{Y}^{marginal}_{ij}(t)= & \sum \limits _{l=1}^L X_{ij,l}\widehat{\beta }_l(t) \nonumber \\ \widetilde{Y}^{conditional}_{ij}(t)= & \sum \limits _{l=1}^L X_{ij,l}\widehat{\beta }_l(t) + \widehat{\alpha }_{i}(t). \end{aligned}$$

We define a global estimator of $R^2$ for the FoSR and MFPCA models as the integral of the point-wise $R^2$ estimates over the time interval $t \in [0, 360]$. Since the data are observed on a discrete grid, this integral is approximated using a numerical quadrature formula, i.e. as a weighted sum of the point-wise $R^2$ values with integration weights $w(t),t \in T_m$. We employ simple trapezoidal weights for this purpose. The resulting estimator is summarized in Eq. 10:

10 $$\begin{aligned} \widetilde{R}^2 = \frac{1}{360} \int _{0}^{360} \widetilde{R}^2(t) dt \approx \frac{1}{360}\sum \limits _{t \in T_m} w(t)\widetilde{R}^2(t). \end{aligned}$$

## Results

### Multilevel functional principal components analysis

**Fig. 3:**
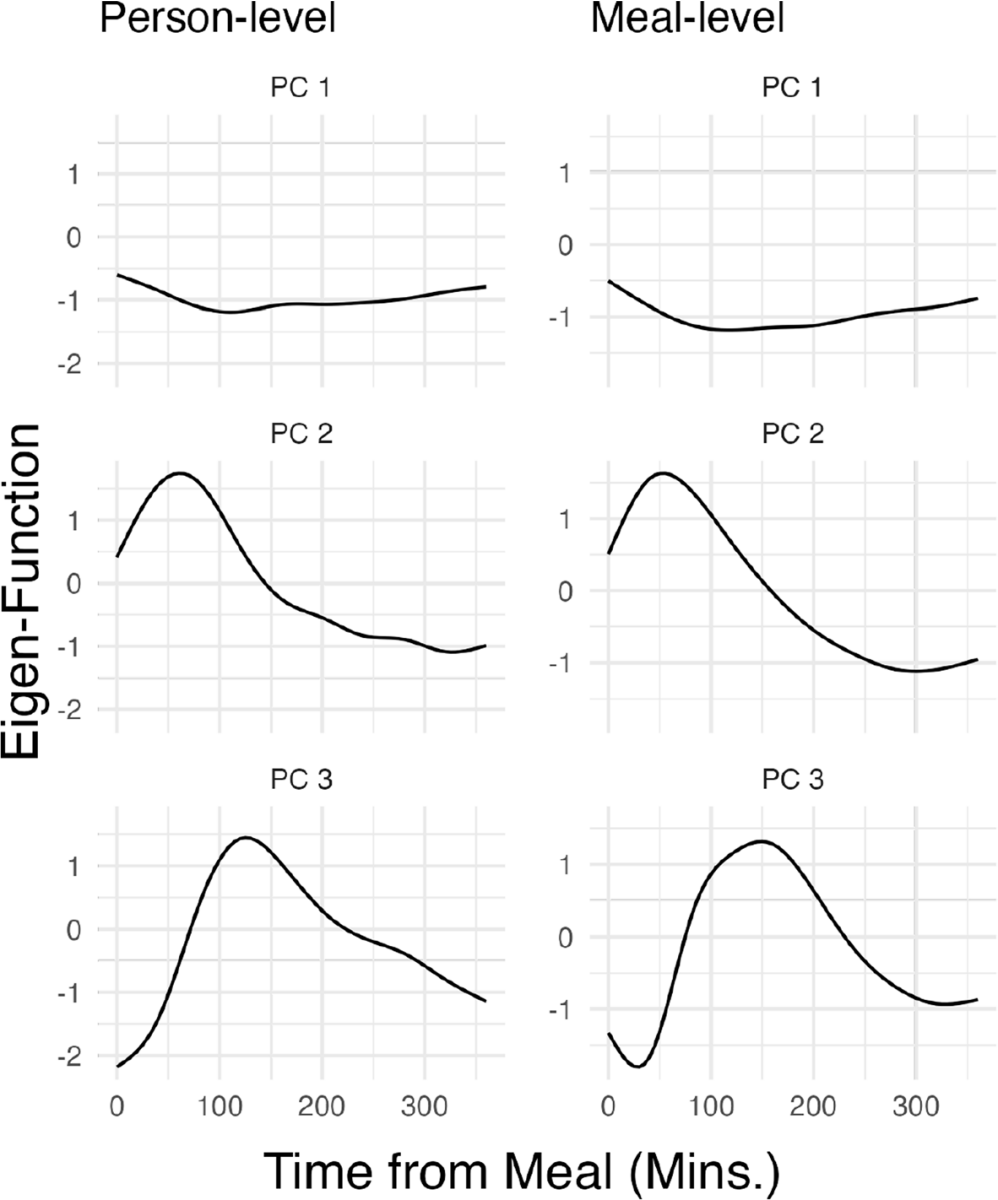
Eigenfunctions of the postprandial CGM responses at the subject and meal levels

The purpose of this section is to apply multilevel functional principal components analysis (MFPCA) to the postprandial glucose trajectories from AEGIS participants without diabetes to understand the modes of variability in the post-meal response over time. Figure 3 displays the first three eigenfunctions at the person and meal levels, that is, the three orthogonal functions that account for the most variability in $U_i(\cdot )$ and $W_{ij}(\cdot )$, respectively. The first two eigenfunctions at the individual and meal levels in Fig. 3 captured a substantial portion of the total variability (more than 80%). The first eigenfunctions at both levels suggested an almost time-invariant absolute level, with a relatively shallow concavity peaking at around 100 minutes. The second eigenfunctions contained a more pronounced peak around 60–80 minutes after the meal. There is also a noticeable similarity of the eigenfunctions across the two hierarchical levels.

Recall that MFPCA provides functional data representations that summarize all trajectories as linear combinations of eigenfunctions weighted by scores. The first eigenfunction is the functional direction that explains the highest proportion of variability. The first eigenfunction, which is close to a constant function with slight curvature around 100 minutes, indicates that the main difference in postprandial responses, both between and within participants, is the mean glucose level. In addition, individuals with higher mean levels exhibit slightly greater concavity around 100 minutes. This suggests a correlation between higher postprandial responses and later peak glucose, which aligns with the physiological understanding of how variation in diet and individual capacity to secrete and respond to insulin can affect glucose processing [18].

The second and third eigenfunctions at both levels can be interpreted in a physiological context: (2) how high the postprandial peak is (at approximately 60 minutes), with the elimination speed of glucose scaling accordingly to maintain the overall processing time; and (3) whether the glucose peak occurs later, at approximately 100 minutes rather than 60 minutes. Both of these features can be related to higher glycemic index meals or impaired glucose processing in normoglycemic individuals.

**Fig. 4:**
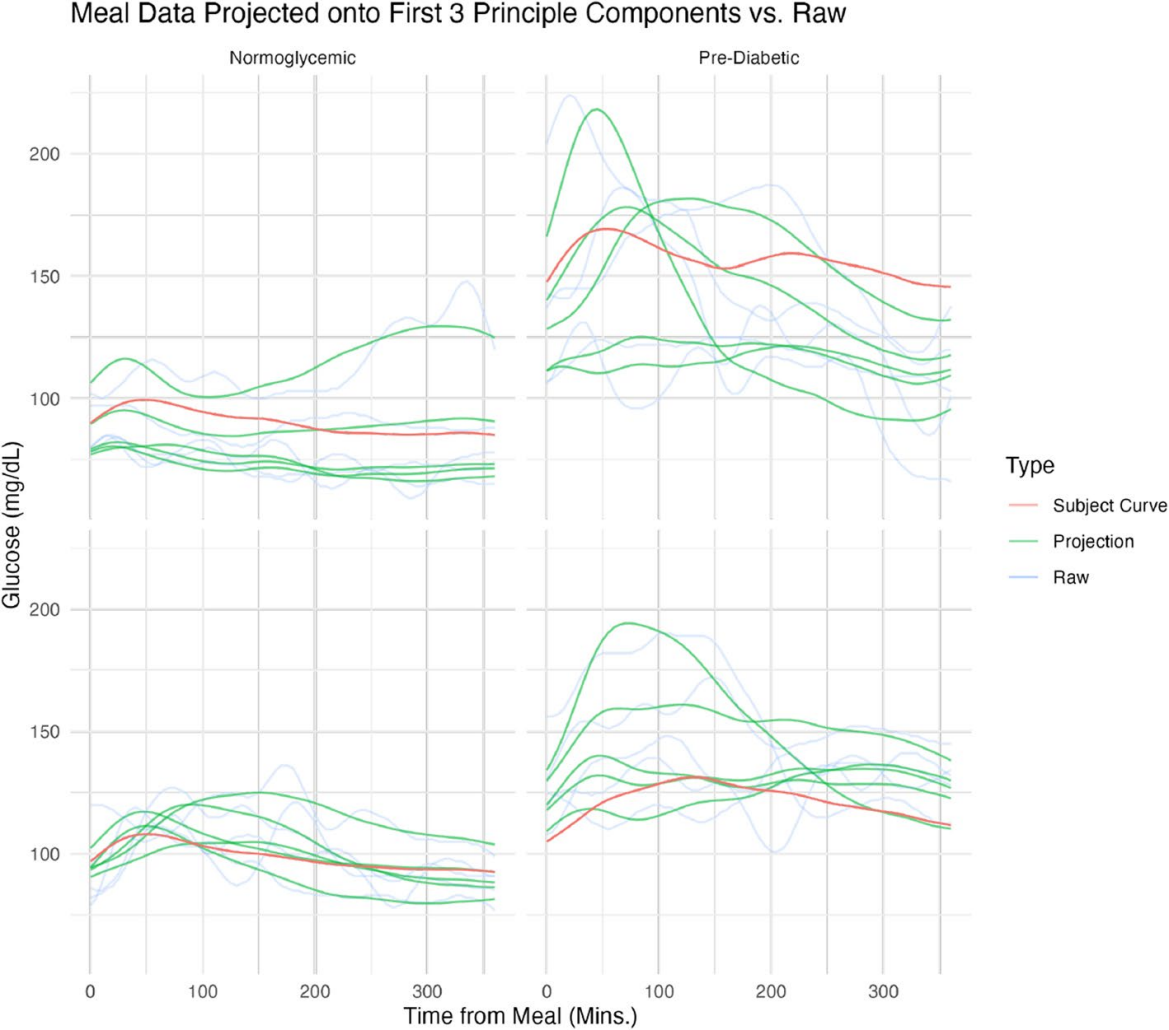
Projections of Raw Data onto Eigen-Functions. Raw Trajectories (Transparent Blue): These trajectories display the original data collected over consecutive days. Estimated Participant Trajectories (Red Curve): Utilizing the scores and eigenfunctions at the individual level, these visualizations illustrate the estimated participant-level trajectories. Projected Trajectory for Each Meal (Green Curves): Constructed from the scores and eigenfunctions at both levels, these curves represent the estimated meals using the parsimonious MFPCA representation

Figure 4 shows the CGM raw trajectories for four randomly selected individuals - two with prediabetes and two who are normoglycemic - along with their corresponding person-specific and meal-specific projections according to the MFPCA model. These components are found using the relevant estimated components from Eq. 1.

We observe pronounced heterogeneity among the projected data at both levels, with variability between and around the subject-specific trajectories. The eigenvalue analysis indicates that, of the variability explained by this MFPCA model, 33% comes from the subject level and the remaining 67% from the meal level, further supporting the hypothesis of important variability existing at both levels. This finding underscores the necessity of using multilevel models to account for CGM data structure.

### Predicting clinical outcomes using latent representations

We applied MFPCA to the residuals of the FoSR model, using person-level eigenfunction scores to assess whether postprandial CGM information can predict the longitudinal evolution of HOMA-IR. HOMA-IR is a key metabolic indicator, closely linked to insulin resistance and progression from prediabetes to diabetes in individuals with obesity. Supplementary Material Appendix A shows the substantial relative improvement in explained variability achieved by incorporating the first two scores into a linear regression model for this continuous biomarker, quantified using the standard coefficient of determination $R^2$. The increase in predictive capacity is more pronounced among participants with prediabetes, despite the much smaller sample size of this subgroup within AEGIS, likely due to the strong biological relevance of HOMA-IR to the progression of diabetes in individuals with impaired glucose regulation or elevated risk of diabetes.

### Function-on-scalar regression

We applied the Function-on-Scalar Regression model to examine the time-dependent association between covariates of interest and postprandial glucose response. The covariates included those detailed in Table 1 and the initial glucose concentration. This glucose value, measured 5 minutes before the recorded meal, was added both to account for the relatively high autocorrelation in CGM data due to the nature of glucose dynamics and to introduce some information related to glycemic condition when the meal begins.

**Fig. 5:**
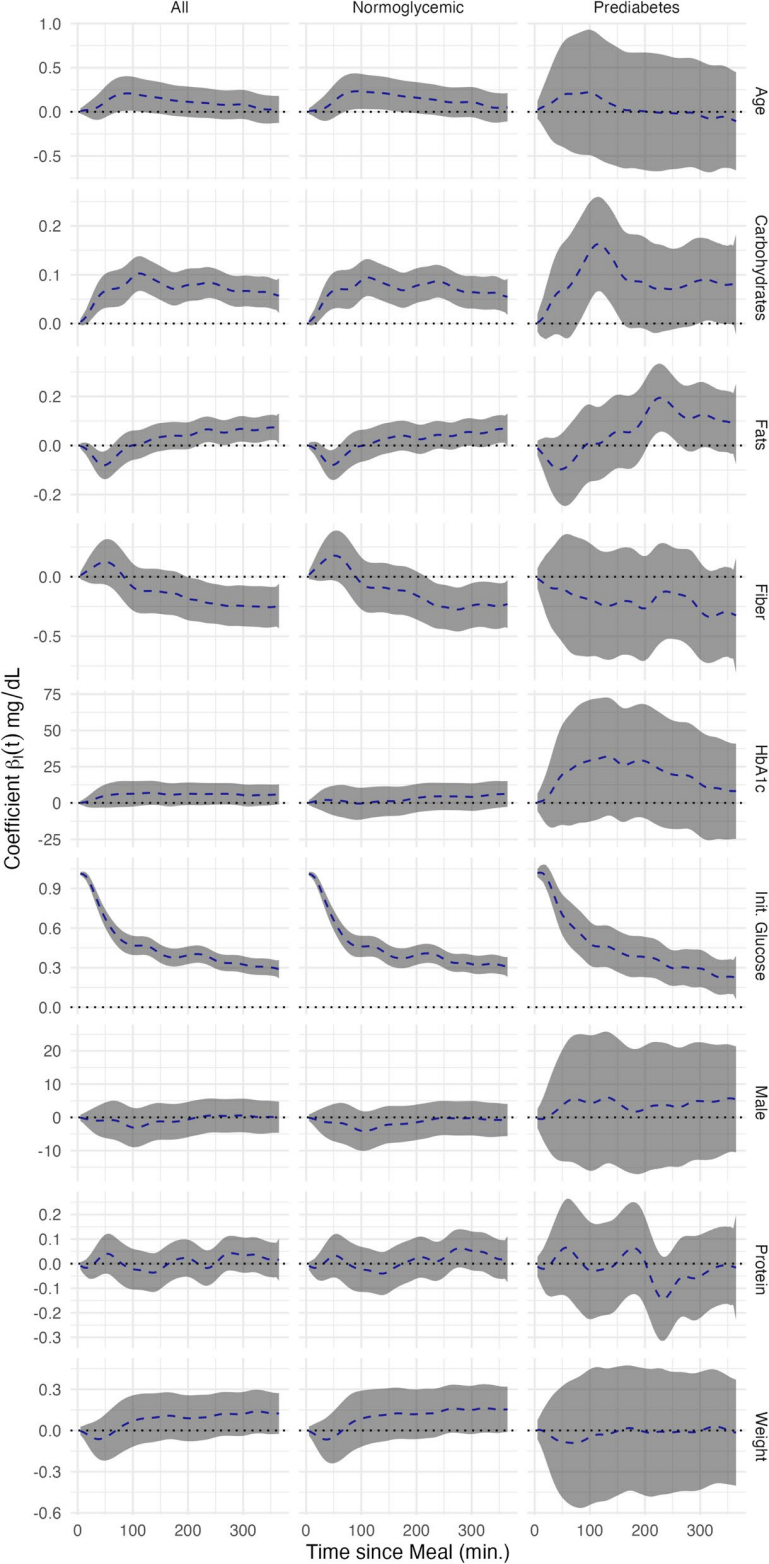
Estimated time-varying covariate coefficients $\widehat{\beta }_{s}(t)$ for each predictor from the multilevel function-on-scalar regression model used to predict postprandial glucose trajectories. Dashed blue curves show the point estimates, grey shading denotes the 95% confidence bands, and the dotted horizontal line at 0 marks the null effect

Figure 5 displays the fixed effect coefficient functions with associated confidence intervals. The first column indicates the coefficient functions for the entire population without diabetes ($n=377$), the second includes only those labeled normoglycemic ($n=319$), and the final column contains those individuals with prediabetes ($n=58$). Each plot contains a dotted line at zero to make it easier to discern where statistical significance is achieved. As Fig. 5 clearly shows, most covariates achieved point-wise significance over some interval in at least one population, but each had a unique coefficient function shape and subsequent interpretation. We did not standardize the predictors prior to fitting FoSR, as we wish to retain interpretability on the natural scale, and comparison between predictors is not the primary goal.

Examining Fig. 5, it is first apparent that the estimates within the population with pre-diabetes were more variable. This was logical given the smaller size of this subset and the greater heterogeneity in glycemic regulation. With increasing age, there was an increase in postprandial glucose concentrations that reached a peak at 90 minutes. This effect gradually declined until it disappeared 5–6 hours after ingestion. No significant differences were observed between men and women. Increased levels of HbA1c were associated with increased glucose concentrations along the continuum from normoglycemic to prediabetes, but not within groups. Higher amounts of carbohydrates corresponded to a substantial increase in the postprandial glycemic response. This effect is magnified in participants with prediabetes, possibly due to lower insulin sensitivity within this group. An opposite effect was observed in meals with higher amounts of fats, where initially (up to 50 minutes after meal) there is a decrease in glucose concentrations, then a slow and mild increase in glucose concentration. Again, this effect was greater in prediabetic individuals than in normoglycemic individuals. Protein intake did not alter post-dinner glucose concentrations; the confidence-band analysis did not show statistically significant effects throughout the functional domain. In contrast, increasing fiber appeared to attenuate the glycemic response about 90 minutes after the post-dinner glucose peak, significantly lowering glucose levels in normoglycemic individuals. No significant time-dependent effects were observed in participants with prediabetes, although the point estimates suggested a reduction in glucose values throughout the domain. The initial blood glucose concentration was highly significantly associated with the postprandial response, being particularly influential in the time directly after the meal. Although this could be due to the high temporal autocorrelation in the CGM data, the observed effect did not decay to zero over the course of the meal window. The higher initial glucose concentrations thus seemed to indicate postprandial glucose concentration in more than an auto-regressive capacity.

### $R^2$ for multilevel functional models

Next, we assessed the variance explained by our hierarchical functional models using a newly introduced notion of functional $R^{2}$, defined from both point-wise and global perspectives. Specifically, we computed the values $R^{2}$ for the MFPCA and FoSR models to quantify the proportion of variance explained at each time point $t \in [0,360]$ of the post-prandial period. Defining a functional $R^{2}$ in this context serves two purposes: (i) to determine how much variability is explained by the multilevel functional model–either with or without random effects, and (ii) to guide model specification, for example by selecting the number of eigenfunctions in MFPCA according to the proportion of variability they capture or by deciding whether to include random effects in the regression setting.

We evaluated $R^{2}(t)$ values for the MFPCA model with $K = H = 3$ principal components. The corresponding curves are shown in Supplement 7. These curves reveal that the participant–level models exhibit relatively low explanatory power, highlighting substantial intra–participant heterogeneity. In contrast, the full model performs well overall, except for reduced explainability during the first hour after the meal and the final hour of the observation window.

Supplement 8 was constructed to demonstrate both marginal and conditional functions $R^2(t)$ for the FoSR model. For normoglycemic participants, conditional and marginal $R^2(t)$ values aligned closely in the first 50 minutes, indicating minimal influence of random effects during this period. Later, $R^2(t)$ values stabilized with random effects contributing to a greater than 50% increase in variability explained. In prediabetic individuals, conditional and marginal $R^2(t)$ diverged earlier in the postprandial period, possibly a result of the smaller sample of participants with prediabetes, which also naturally has increased heterogeneity in the glycemic response. In general, the predictive capacity of the model is moderate. In particular, after 90 minutes, the conditional and marginal versions of $R^2(t)$ fall below 0.5.

## Discussion

This paper introduces an FDA framework for studying postprandial CGM responses and demonstrates the application of this framework to the AEGIS study. This modeling strategy semi-parametrically estimates time-varying relationships between predictors and the CGM response while still accommodating participant-specific random effects. To our knowledge, it is the only FDA-based framework in the postprandial glucose literature that incorporates random effects. The FDA models, augmented with the notion of functional $R^2$ introduced in this article, provide two key insights in the context of AEGIS: (1) the importance of accounting for participant-specific random effects; and (2) the estimated impacts of diet and participant characteristics on the trajectories of postprandial glucose response measured by CGM.

There is a large body of literature modeling glycemic responses to food intake. These models are most often based on systems of differential equations governing the various components of the glycemic system [25–29, 32, 50–53]. There are very few such methods that can comprehensively model the glucose time series data from CGM in isolation of insulin and the other hormones that dictate glucose dynamics. Among such models on glucose, the FDA approach is unique in that it is fully data-driven, capable of handling hierarchical structure and estimating semi-parametric, time-dependent associations with scalar predictors (the coefficient functions $\beta _l(t)$). Furthermore, the FDA methods we introduce are computationally scalable and can be applied to large medical-cohort studies, including those currently underway in Israel and the United States [54, 55].

Applying FDA to AEGIS data yields statistically significant, time-varying associations between diet composition and postprandial glucose response. These results were differential according to prediabetes status. Importantly, we observed a differential glycemic response to increased fat intake between normoglycemic participants and prediabetic participants. Further scientific findings discussed in this section are outlined in Table 2. The dietary findings of this study indicate the potential for greater glucose control by combining a better understanding of the dietary impact on postprandial glucose and personalized nutritional recommendations.

**Table 2:** Summary of findings

Result	Implication
There is substantial heterogeneity in mean value and shape of postprandial glucose time series both between and within subjects	Appropriately accounting for the hierarchical structure of the postprandial responses is required for adequate explanation of the observed glucose patterns
The coefficient functions$\beta _l(t)$for different macro and micro-nutrients are not time-invariant, and they vary in intensity and direction.	Shape and mean value of postprandial glucose response are influenced by the composition of macro and micro-nutrients in distinct ways.
The coefficient functions$\beta _l(t)$differ between normoglycemic and prediabetic individuals.	Metabolic responses to the same diet differ between normoglycemic and prediabetic patients, indicating the importance of glycemic capacity in formulating diet recommendations.
The$R^2$functional mixed model explainability metrics are not time-invariant, showing a decline over time, and random effects significantly increase the variability explained in the predictions 50 min after post-meal intake.	Post-meal functional response analysis indicates significant individual heterogeneity, necessitating alternative, perhaps more personalized, model structures.

The FoSR models fit here explain only a moderate proportion of the variability beyond the first hour, even after incorporating the participant-specific random effects. This suggests the presence of latent structure that is not captured by the covariates we collected. We hypothesize that unmeasured factors such as physical activity and details of proximal snacking (timing and composition) contribute to this residual variability. Capturing a larger share of the variance may therefore require adding additional relevant variables or adopting a more flexible random-effects structure. We also emphasize that modeling the postprandial response is intrinsically challenging, largely because of the considerable inter–individual metabolic variability.

Regarding FoSR model assessment and potential over-fitting, we note that the underlying specification remains linear. This simplicity provides an intrinsic safeguard against over-fitting. Linear models are generally robust, though they can be sensitive to mis-specification because of their rigid functional form. The variability observed among participants with pre-diabetes reflects the true inter-individual heterogeneity within this subgroup and the fact that we analyzed a relatively small subsample ($n = 58$). As future work, we plan to apply more flexible functional models that can capture general nonlinear, time–dependent associations between diet and glucose trajectories. These methods, which range from advanced statistical approaches to machine-learning methods, should also carefully balance the increased risk of over-fitting.

In this paper, we focus on the fui function because it provides inference comparable to the state-of-the-art functional methods while remaining computationally lightweight, allowing the analysis of thousands of CGM curves in seconds. For smaller sample sizes ($n < 30$), where inference is more sensitive, a one-step multilevel functional model implemented via fosr, or Bayesian approaches that incorporate expert knowledge through informative priors may be preferable.

A primary strength of this particular FDA application to the AEGIS study is the generalizability of the results. The AEGIS trial includes a random sample of the general Spanish population, and the normoglycemic sub-group is of sufficient size. The results in this group should generalize well to similar populations.

The FDA techniques described here can be applied to CGM data from a wide range of populations. Extending the methods to cohorts comprising individuals with normoglycemia, pre-diabetes, or non–insulin-treated type 2 diabetes is straightforward. However, in groups with type 1 diabetes or insulin–treated type 2 diabetes, it is essential to incorporate participant-specific treatment regimens, particularly the timing of insulin administration. Once these factors are properly modeled, FDA will produce the same depth of characterization and insight that we achieved for the AEGIS cohort.

## Conclusion

Our FDA-based approach to modeling postprandial CGM data provides a method to estimate the time-varying associations between dietary/other factors and PPGR, while accounting for the hierarchical structure of multiply-observed data. We extend the general modeling framework by introducing an adaptation of the traditional measure $R^2$ to the case of multilevel functional models. The corresponding results indicate the key importance of including person-specific random effects when modeling these data, as well as the room for improvement even after accounting for these effects. With the increasing prevalence of large cohort studies that include wearables data from devices such as CGMs, actigraphs, and patch electrocardiograms (ECG), this framework provides a core set of tools for analyzing and characterizing the corresponding multilevel functional data.

For future work, we propose extending the concept of CGM-based phenotypes, such as the eminent glucotypes [17], using the multilevel functional (regression) modeling framework.

## Figures and Tables

**Fig. 6:**
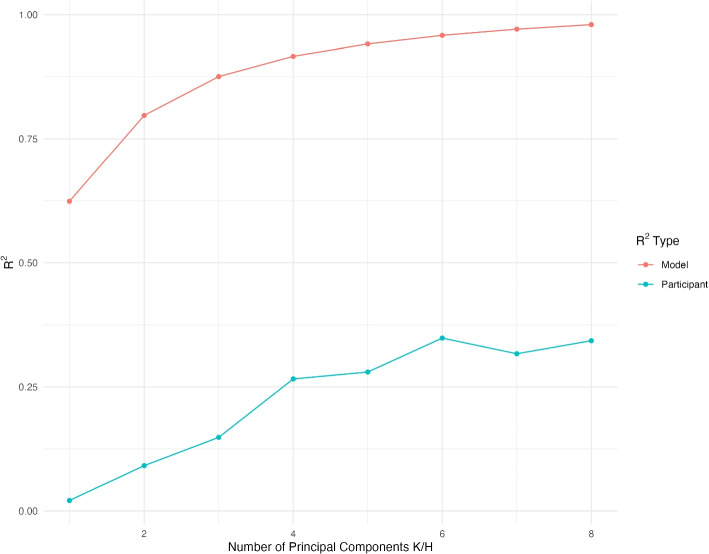
Participant-level and model-level global $R^2$ estimates for MFPCA models by number of eigenfunctions *K*, *H* at both levels

**Fig. 7:**
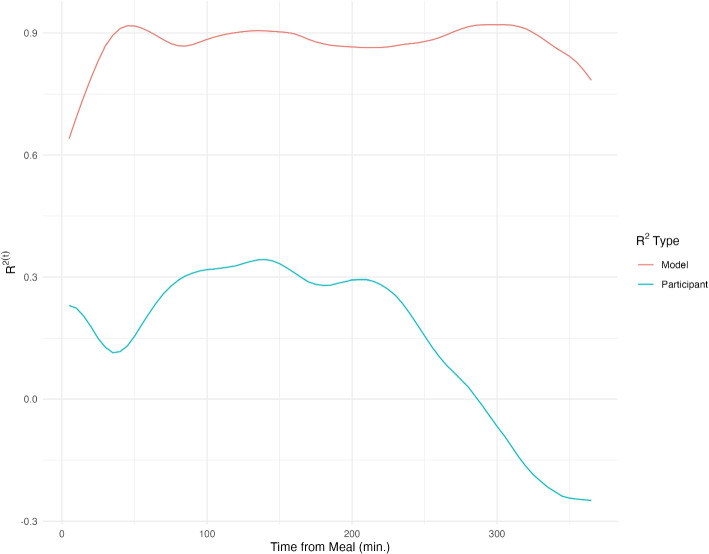
Participant-level and model-level $\widetilde{R}^2$ estimates for the chosen MFPCA model with $K = H = 3$ eigenfunctions at both levels

**Fig. 8:**
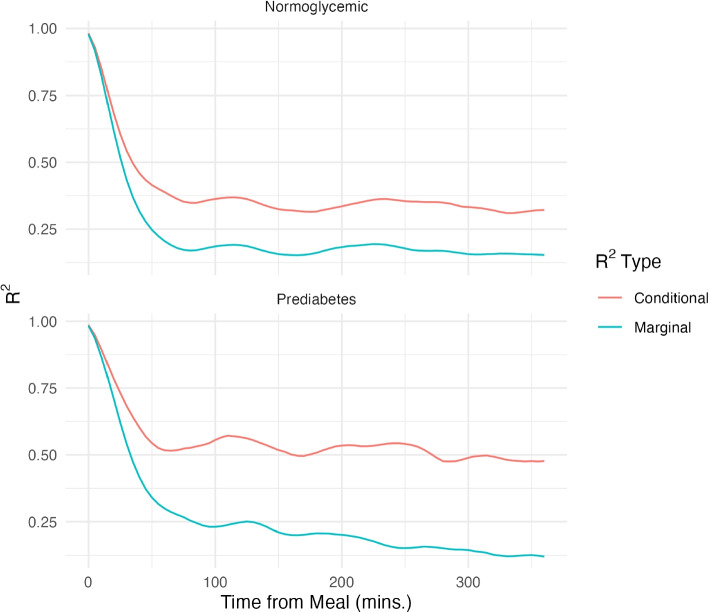
Marginal and Conditional $R^2$ plotted over the course of the postprandial observation window

## Data Availability

Some Subset of the data is public.

## Abbreviations

CGM: Continuous glucose monitoring
AEGIS: A Estrada Glycation and Inflammation Study
FDA: Functional Data Analysis
MFPCA: Multilevel Functional Principal Components Analysis
FoSR: Function-on-Scalar Regression
ECG: Electrocardiogram
